# Better together? a naturalistic qualitative study of inter-professional working in collaborative care for co-morbid depression and physical health problems

**DOI:** 10.1186/1748-5908-8-110

**Published:** 2013-09-20

**Authors:** Sarah E Knowles, Carolyn Chew-Graham, Nia Coupe, Isabel Adeyemi, Chris Keyworth, Harish Thampy, Peter A Coventry

**Affiliations:** 1 NIHR School for Primary Care Research, Manchester Academic Health Science Centre, University of Manchester, Manchester, M13 9PL, UK; 2 Primary Care and Health Sciences, Keele University, Keele, Staffordshire, ST5 5BG, UK; 3 Greater Manchester Collaboration for Leadership in Applied Health Research and Care, Institute of Population Health and Manchester Academic Health Science Centre, University of Manchester, Manchester, M13 9PL, UK

**Keywords:** Depression, Mental health, Co-morbidity, Implementation, Qualitative, Collaborative care, Chronic care, Primary care

## Abstract

**Background:**

Mental-physical multi-morbidities pose challenges for primary care services that traditionally focus on single diseases. Collaborative care models encourage inter-professional working to deliver better care for patients with multiple chronic conditions, such as depression and long-term physical health problems. Successive trials from the United States have shown that collaborative care effectively improves depression outcomes, even in people with long-term conditions (LTCs), but little is known about how to implement collaborative care in the United Kingdom. The aim of the study was to explore the extent to which collaborative care was implemented in a naturalistic National Health Service setting.

**Methods:**

A naturalistic pilot study of collaborative care was undertaken in North West England. Primary care mental health professionals from IAPT (Increasing Access to Psychological Therapies) services and general practice nurses were trained to collaboratively identify and manage patients with co-morbid depression and long-term conditions. Qualitative interviews were performed with health professionals at the beginning and end of the pilot phase. Normalization Process Theory guided analysis.

**Results:**

Health professionals adopted limited elements of the collaborative care model in practice. Although benefits of co-location in primary care practices were reported, including reduced stigma of accessing mental health treatment and greater ease of disposal for identified patients, existing norms around the division of mental and physical health work in primary care were maintained, limiting integration of the mental health practitioners into the practice setting. Neither the mental health practitioners nor the practice nurses perceived benefits to joint management of patients.

**Conclusions:**

Established divisions between mental and physical health may pose particular challenges for multi-morbidity service delivery models such as collaborative care. Future work should explore patient perspectives about whether greater inter-professional working enhances experiences of care. The study demonstrates that research into implementation of novel treatments must consider how the introduction of innovation can be balanced with the need for integration into existing practice.

## Background

Depression is estimated to be two to three times as common in people with long-term conditions (LTCs) and negatively impacts on medical management of disease and self-care behaviors, leading to poorer quality of life and high costs in primary care. However despite initiatives to encourage detection of depression in patients with co-morbid physical illness, people with LTCs and depression are less likely to receive treatment than people with depression alone [1]. Co-morbidities pose significant challenges both for patients [2] and for the services that care for them [3]. Such challenges have been viewed as the result of healthcare systems which have historically focused on disease-specific protocols, rather than managing care across multiple chronic conditions, with physical-mental health co-morbidities particularly at risk of fragmented care [4,5].

In recent years, United Kingdom (UK) health policy for managing LTCs has been informed by United States (US) approaches to quality improvement and service redesign: the chronic care model and the ‘risk pyramid’ developed by Kaiser Permanente [6]. These US models are underpinned by a philosophy that appeals to whole system perspectives in which healthcare systems are seen as the main barrier to delivering effective treatments for LTCs. The chronic care model emphasises that care improvement is linked to the redesign of inter-dependent components in health systems: delivery system design; patient-provider relationships; decision support tools; clinical information systems; community resources; and organizational factors, such as leadership.

Over the last decade evidence has accumulated to show that adoption of the principles and practices of the chronic care model can lead to improved depression care. In particular, the chronic care model has underpinned the development of collaborative care which includes both organizational and patient-level intervention components designed to facilitate the delivery of treatments of varying intensity based on evidence based stepped care treatment algorithms and consists of four key components [7]:

### Components of a collaborative care intervention (Gunn *et al.* 2006)

1. 1. A multi professional approach, requiring a general practitioner/family physician plus at least one other health professional.

2. 2. A structured management plan.

3. 3. Scheduled patient follow ups.

4. 4. Enhanced inter-professional communication.

There is now robust evidence that collaborative care is more effective than usual care for treating depression [8], reinforcing the finding that interventions that address how to improve service integration and co-ordination between health professionals are more effective than clinical guidelines or education alone [9].

In the US, collaborative care has also been shown to improve depression in people with LTCs [10,11]. However, despite the growing evidence base for collaborative care, there remains a gap between the demonstrated efficacy of collaborative care in trials and its implementation in everyday practice [12,13]. This is particularly true in the English National Health Service (NHS) where the National Institute for Health and Care Excellence (NICE) have recommended using collaborative care for patients with depression and LTCs where mental health status has not improved as a result of medication and/or a high intensity intervention. However, NICE have not fully identified and defined key components of successful collaborative care for depression and LTCs. Additionally, in the context of LTCs, there is scope to identify optimal care pathways associated with collaborative care for managing patients with both physical and mental health problems. To this end, trials are now underway to test the effectiveness of collaborative care models for people with LTCs in primary care settings outside of the US.

As part of a wider program of research about improving the quality of care for people living with chronic vascular disease, the Greater Manchester CLAHRC began a randomized controlled trial in the North West of England (COINCIDE; http://clahrc-gm.nihr.ac.uk/coincide/) of collaborative care for depression in patients with diabetes and/or coronary heart disease [14]. CLAHRCs are explicitly focused on addressing the second translational gap identified in the Cooksey (2006) report. While the ‘first gap’ in translation concerns the translation of basic research into clinically meaningful outputs, the ‘second gap’ concerns the translation and integration of this clinical knowledge into actual practice. Closing the second gap therefore demands greater emphasis on understanding and evaluating the implementation of innovative treatments that are likely to be effective and appropriate for use in routine care. As part of the UK government’s commitment to mainstreaming mental health services into primary care and reducing inequities in access to physical and mental healthcare, the Improving Access to Psychological Therapies program (IAPT) is committed to broadening the benefits of talking therapies to people with LTCs [15]. To meet the strategic goals of the CLAHRC to evaluate implementable interventions and to map research goals to service commitments in the NHS, the COINCIDE trial therefore developed a training package to support for IAPT workers to deliver collaborative care for people with depression and LTCs to explore whether collaborative care can improve access to depression care for people with LTCs and depression of varying severity and in settings beyond the US.

The UK Medical Research Council has developed a framework for evaluating complex interventions, which emphasizes the need to examine both barriers and facilitators of implementation [16]. In keeping with the MRC Framework, the COINCIDE trial piloted the collaborative care intervention in the pilot phase described in this paper.

### Collaborative care pilot phase: implementation in a naturalistic setting

The collaborative care pilot was undertaken in a large Primary Care Trust in North West England. In England, psychological therapy for people with common mental health problems is typically provided for by psychological well-being practitioners (PWPs) employed by IAPT (http://www.iapt.nhs.uk/). PWPs are usually graduate psychologists and provide high-volume, low-intensity psychological interventions based on a cognitive and behavioral model for patients with depression and anxiety disorders. In existing services, PWPs are therefore involved exclusively in treatment for mental health problems and provide a stand-alone service based on referrals from general practitioners (GPs), rather than in collaboration with other practice staff. In the collaborative care pilot, six PWPs were trained as case managers to provide brief psychological interventions for depression in patients with diabetes, coronary heart disease (CHD) and chronic obstructive pulmonary disease (COPD). Case managers in collaborative care are intended to act as ‘conduits’ between patients and primary and specialist care providers. Although US models of collaborative care have employed nurses as case managers to liaise between GPs and patients, the COINCIDE trial employed PWPs as case managers given that practice nurse consultations in the UK are heavily structured and driven by the need to meet quality improvement targets which could limit the nurses capacity to engage fully with collaborative care activities [14]. Core training therefore consisted of the following:

### Training for health professionals in collaborative care for patients with LTCs

Core training for PWPs in the Pilot Study (five days):

•Understanding LTCs (Diabetes, COPD, CHD)

•Interventions to manage depression, anxiety and LTCs

•Medication management and lifestyle interventions

•Behavioral activation and cognitive interventions for LTCs

•Effective liaison with practice staff

Core training for Practice Nurses in the Pilot Study (1 day):

•Talking about anxiety and depression with patients with LTCs

•Formal assessment and screening of anxiety and depression in patients with LTCs

•Low intensity interventions for anxiety and depression – introducing the interventions to patients

•Implementing the care pathway

**Figure 1:**
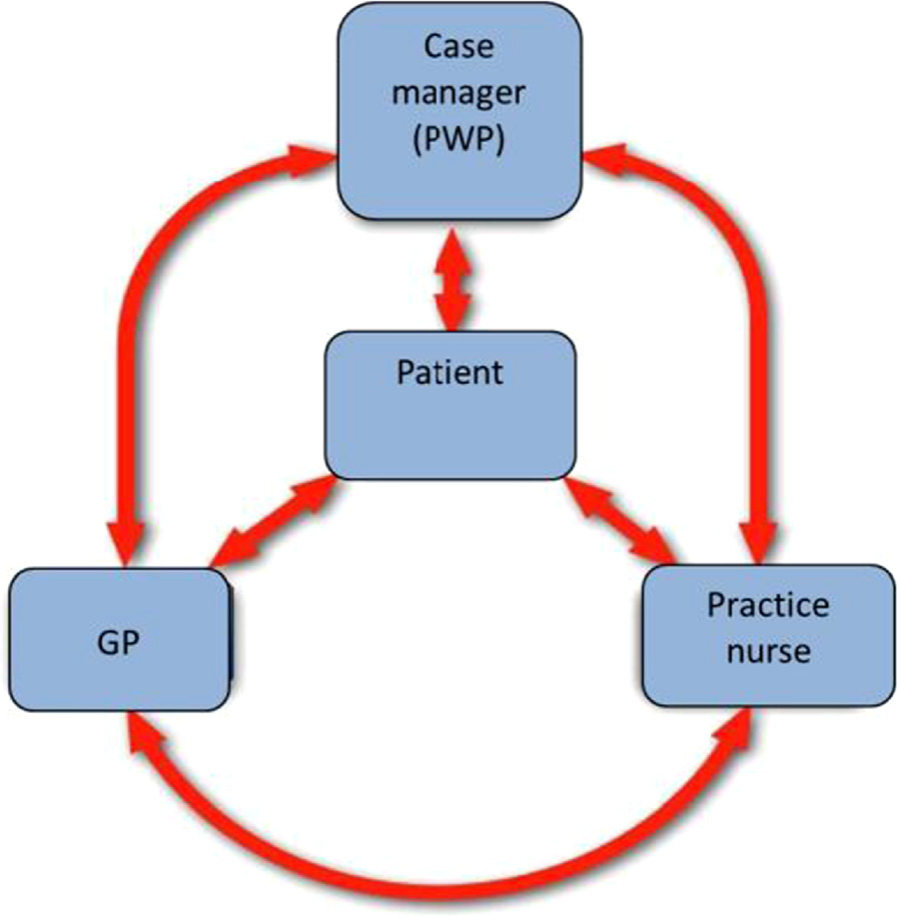
Case management in the COINCIDE Trial.

Practice nurses, employed by general practices in the NHS trust, also participated. In the UK, practice nurses are central to chronic disease management and coordinate care between the GP and the patients, and consequently the COINCIDE trial focused on collaboration between practice nurses and PWPs rather than with GPs, given the aim of CLAHRC to evaluate interventions most likely to be implemented in routine settings (although GPs were still considered to be part of the collaborative care model given that they retain overall responsibility for the patients). A full discussion of the rationale for focusing on nurses and PWPs is given in the trial protocol [14]. The training included skills development to identify depression in patients with LTCs attending routine physical health monitoring appointments and facilitate referral of patients to treatment with the PWPs based in the practice (Figure 1). Training also recommended explicit collaborative working between the PWPs and practice nurses, including holding joint consultations with patients (Box 2).

### Theoretical model of implementation: normalization process theory

Employing theoretical models of implementation is recommended to enable researchers to identify the conditions necessary for interventions to be successfully adopted in routine primary care [17]. Normalization Process Theory (NPT) offers a set of conceptual and explanatory tools to understand how innovations, such as collaborative care, become embedded in everyday work. It has been successfully employed to evaluate how innovations in depression care have been deployed and integrated in primary care [18-20]. As the pilot study was run under naturalistic conditions, this presented a unique opportunity to explore the extent to which collaborative care was implemented in routine primary care, potentially leading to better understanding about barriers and challenges associated with translation of complex interventions outside of trial settings. The aims of the study were to:

1. Explore the extent to which these collaborative care principles and modes of working were implemented in routine care for the management of patients with depression and exemplar LTCs (diabetes, CHD, COPD),

2. Employ NPT as a conceptual model to identify barriers and facilitators to the adoption and integration of collaborative care in routine practice.

## Methods

Ethical approval for the study was received from Greater Manchester West REC.

### Design

Longitudinal qualitative exploration to examine early implementation at three months (time one – T1) and reflections on implementation at the end of the nine-month pilot (time two – T2). Practitioners were interviewed at T1 to explore early adoption of the intervention and understanding of the collaborative care model, and at T2 to explore the health professionals’ reflections on the effectiveness of the model and its impact on patient care and inter-professional working. Face to face semi-structured interviews were performed to explore perceptions and experiences of collaborative care in practice. All interviews were recorded and transcribed verbatim.

### Sample

Six PWPs who had been trained as case managers and 17 practice nurses (PNs) were approached for interview. At T1, all PWPs and 12 PNs took part in interviews, representing nine practices in total. At T2, five PWPs and seven PNs took part.

### Analysis

A preliminary thematic analysis using the constant comparative method identified primary themes across the data set, which was compared with the NPT constructs to determine its applicability. Transcripts were read independently by the authors and labels and themes compared to ensure the credibility of key themes. Analysis was repeated at T2 through consensus meetings with all authors, to check for divergent or novel themes and consider the persistence of the original themes over the duration of the pilot phase. Constructs and potential examples are outlined below.

### NPT Constructs (May and Finch 2009)

1. **Coherence**: The meaning of the practice to actors – is there agreement on what the work is?

e.g., Is work on depression viewed as consistent with treating LTC? Is there shared understanding of what needs to be done to address co-morbidities?

2. **Cognitive participation**: engagement, individually and collectively, with the practice – is there agreement about who does the work?

e.g., What kind of norms exists around who should carry out interventions? Is there ‘buy in’ to the procedures, and the integration of physical and mental healthcare, by all actors?

3. **Collective action**: Interaction with pre-existing or established processes - is there agreement about how the work gets done?

e.g., Is there formal or informal agreement about what works need to be done to achieve collaboration and what activities need to be performed to do it?

4. **Reflexive monitoring**: how the practice is assessed and understood by the actors – is there agreement on how to appraise the work?

e.g., How is the collaboration monitored? Is there the necessary infrastructure and resources to enable review between professionals?

## Results

### Primary thematic analysis at T1: experience of collaborative care

Preliminary themes from the thematic analysis represent two broad constructs: ‘Coming together’ and ‘Staying apart’ (Table 1). These themes relate specifically to perceptions about how the collaborative care model encouraged novel ways of working that were distinguishable from working practices typically seen in primary care where PWPs and PNs work separately, both geographically and in terms of therapeutic focus.

**Table 1:** Barriers and facilitators to the collaborative care model

**Coming together**	**Staying apart**
**Organizational facilitators:**	**Organizational barriers:**
**Co-location:**	**Lack of integration:**
• **Allows informal collaboration:** ‘Last week there was a question that I had about somebody’s diabetes and the use of insulin… I actually just popped my head around the door for one of the practice nurses…just being there meant that I could ask her that.’ PWP01	• **Lack of shared resources ; time for appointments not allocated by practice managers, limited access to practice information systems:** ‘ ‘They’ve [nurses] got so many other things to factor into their appointments, they forget that I’m there.’ PWP05
• **Destigmatises access to mental healthcare:** ‘It’s based in the surgery…it’s not something strange and new and it works well, being in the same building, definitely, than going somewhere else.’ PN10	‘I feel quite blind by not having [access to] that [IT] system.’ PWP04
	• **GPs unaware of or uninvolved with the PWPs:** ‘GP’s don’t even, I don’t think they know what the [IAPT] service does, never mind the role of the [PWP] practitioner.’ PWP02
	‘GPs… they’re obviously not doing it themselves… They probably haven’t got time, but we haven’t either.’ PN11
**Attitudinal Facilitators:**	**Attitudinal Barriers:**
**Perceived benefit of providing holistic care:**	**Role boundaries:**
• **Increased co-ordination and continuity of care:** ‘Hopefully they’ll [patients] feel that there’s that continuous care kind of thing… they’ll see that it’s sort of a joined up kind of care and that they’re not just put onto another system, and that we’re still all talking to each other.’ PN01	• **Clear division of mental and physical health work and expertise:** ‘You’ve kind of got the mental bit which is me and then the physical which is the nurse…’cause I don’t want the patients thinking that I can help them…and it’s trying to be really clear that that’s not my role.’ PWP02
• **Easier disposal route encourages detection:** ‘Already I’m more enthusiastic about talking about [depression] and approaching it… because I feel I have something to give now… so instead of this skirting around the subject that I started off with, I feel I can go there now and talk about it quite happily’ PNO5	• **Joint meetings perceived as unnecessary:** ‘The nurse comes in just for the last fifteen minutes, otherwise she’d be bored rigid and she has better things to do than listen to me doing my bit.’ PWP02
	• **Lack of confidence to engage in the other area of work:** ‘I feel that they [practice nurses] don’t have enough time to talk to people about their emotional wellbeing, and I think that they worry that if they start talking about it they’ll open Pandora’s Box.’ PWP05
‘I feel a lot more comfortable about doing it and because if people do say anything then I know that I have something I can do about it, can suggest something.’ PN08	

### Normalization process theory: analysis of barriers and facilitators of implementation

NPT proposes four constructs that impact on implementation, concerned with the ‘work’ of engaging with and enacting a practice (Box 3). Examining the themes using NPT revealed a discrepancy between perceptions about the patient-level aspects of collaborative care and the service-level aspects of collaborative care. Specifically, the data showed that coherence and cognitive participation were enacted at the patient level of the model, but not for the professional level, leading to a lack of collective action and reflexive monitoring. This analysis (summarized in Additional file 1) revealed that this division of the ‘work’ of collaborative care about patient and professional components appeared to be driven by the perceived division of mental and physical healthcare.

The participants showed ‘buy in’ for the need to provide more holistic, integrated care and for including psychological treatments in primary care (as shown both in the quotations in Box 1A on Additional file 1, and ‘Coming Together’ in Table 1), but this coherence was still framed around existing norms about the division of mental and physical health work (Box 2A Additional file 1, ‘Staying Apart’ Table 1). Consistent with this separation of mental and physical health at the professional level, there was a lack of coherence and cognitive participation about how to implement the professional level treatment components, including case-management and inter-professional working (Boxes 1B and 2B). Practice nurses did not appear to recognize the collaborative model, and PWPs struggled with their role as a case manager, consistent with the absence of coherence in relation to the collaborative care model itself. This appears to negatively impact on opportunities for collective action and reflexive monitoring across the whole of the pilot (Box 1C and 2C, Additional file 1).

### Analysis at T2: ‘coming full circle’ versus ‘different help’

Interviews at T2 showed persistence of the themes over time and indicated that divisions at T1 were largely maintained. The quotation below illustrates that by the end of the pilot, the separation of collaborative care into a patient level intervention delivered by PWPs and then ‘an addition’ of collaboration with nurses was hindered by both logistical and motivational barriers to joint working:

PWP01:

‘I've understood throughout, I think, what we were set out and what we were kind of commissioned to do, paid to do, what the role is.’

Interviewer:

‘And how about collaborative care, how have you seen that?’

PWP01:

‘Well, that's been an addition really to what I thought the role would be. The collaborative care I think I'm in two minds about….it was set up formally to do session two and session eight. But logistically it was just not happening a lot of the time, because of different kind of diaries and different situations… and not kind of sure how we overlap, or how much the kind of investment and time and energy that we were willing to put into it.’

Both the PWPs and nurses maintained their physical and mental health role boundaries through the duration of the pilot. Even when the benefit of being perceived as part of the practice was recognized, professionals still maintained the division that they are ‘different help’:

PWP01:

‘Some of the good stuff was that when the patients came in, they thought you were part of the GP staff already; they were turning up and seeing it as part of their overall health and care….. The other side of that, [was] when people would ask you stuff that was quite health-based. And you'd be like, okay, how can we do this, because I'm not this person, we're here for different help.’

Practice nurses also continued to view the benefit of having PWPs within the practice as being a more efficient separation of mental and physical healthcare:

PN06:

‘The PWP …took that load off me having to bring patients back on a periodic review just for their mental health. I would allow that for [the PWP] to actually work on, it was pointless duplicating what she might be doing anyway…and, as I say, it’s an advantage from my point of view, because I could actually designate that to her particularly and leave that side of the care to her.’

The PWPs however felt that the pilot would have worked better if the nurses had a better understanding of the work that PWPs did:

PWP02:

‘I think it probably would have been helpful for them to actually get an understanding of what we do. And I know one of them did actually mention that [shadowing the PWP] at the time, but then finished the same sentence with oh but we don’t have time for that.’

Furthermore, this distance was suggested as factor affecting the decision to hold joint meetings, and it was recognized that collaboration itself was a ‘new way of working’ and new skill to be learned:

PWP04:

‘I think there are a lot of reasons why that first joint session often felt really awkward and I think part of that is just because it’s a new way of working for everybody involved and of course the anxieties because, you know, we’re developing our skills as a way of managing these collaborative care meetings, that anxiety and the clunkiness, the client picks up on that and we all feel awkward in that situation.’

Those staff who had repeated later joint meetings however perceived them as beneficial, not only for patients, but for themselves:

PN05:

‘There was no awkwardness because by then [the final joint session] they knew her really well and they've known me for years and years… I think it made it feel like it had come full circle, because it started off with me and it was finishing off with me. And I think they felt that it was all within the comfort of this place where they're used to coming ….I suppose it gave me more insight into what was troubling the patients; patients who I see and with whom I'd never delved or probed. You might tend to concentrate on the clinical. So it's probably taught me more sensitivity and given me confidence to…now that it's [the pilot] finished, it's still given me confidence to broach these subjects.’

## Discussion

Gask *et al.* have commented that the implementation of collaborative care models in the setting of the NHS means that existing relationships, received wisdom about ways of working, and professional roles are challenged [8]. This study demonstrates that inter-professional working around physical and mental health co-morbidities may pose a particular challenge, as health professionals’ perceptions that physical and mental health work are distinct are deeply ingrained, leading to services adopting only limited elements of prototypical collaborative care interventions.

Our findings are consistent with recent evidence from Cheney and colleagues on implementation of collaborative care for depression in the US that shows that adapting research-designed collaborative care for use under naturalistic conditions can be problematic because ‘clinician predispositions’ to adopting the model can vary [21]. Cheney *et al.* reported that patient access to full collaborative care was shaped more by who their provider was than by patient need, concluding that active engagement with collaborative care by primary care professionals is essential. The findings reported in our study demonstrate that similar problems occur in the UK NHS, especially in the context of managing complex physical and mental health co-morbidities. Our findings are also consistent with those of Wells *et al.*[22] who reported that under non-academic managed conditions outside of trials, limited changes to processes of care occur, indicative of the difficulties of introducing service innovation into routine settings.

### Strengths and limitations

This is the first study of collaborative care for mental-physical multi-morbidity in the UK under naturalistic conditions, contributing to the Department of Health’s aim to improve understanding about overcoming the translational gap between research and practice, especially for complex interventions such as collaborative care. The study is further strengthened by the use of a theoretical framework of implementation (NPT) to conceptualize barriers to the provision of collaborative care in routine services.

The main limitation of the study was that data collection was restricted to professionals’ views only, and so it is unknown whether the degree of collaborative working between health professionals had any impact on patient experiences or on outcomes. While data collection on patients was planned, reluctance to refer patients to the research team by the IAPT service made this impossible. Clinical and research priorities were not always aligned, highlighting the difficulties of undertaking evaluations in naturalistic settings where services must be convinced to employ new interventions before the effectiveness of those interventions is established. Models such as NPT stress that the likelihood of implementation be considered during the evaluation phase, rather than as an afterthought. However, financial and resource commitment to deliver a new service may then reduce willingness to fully evaluate the success of the new intervention. Negotiating this tension between service and research agendas will be necessary if projects are to cross the translational gap and develop treatments that are both evidence-based and sensitive to the contextual and organizational needs of service implementation.

A further limitation is that fewer practice nurses participated at the second interview stage. This may be reflective of disengagement with the study over time due to the lack of structured involvement of the study team in this naturalistic pilot. Of a total of 13 practices that took part in the pilot, only nine are represented in the present study and the views of the four that did not agree to take part may be different to those expressed by practices willing to take part. We also did not interview GPs participating in the study and therefore do not know their views on the collaborative working and on the choice of PWPs as case managers. GP interviews in future research will be particularly important to explore their understanding of collaborative care and what collaborative should look like in routine primary care in the UK.

### Implications for research and practice

The divergence from the prototypical model of collaborative care in this naturalistic study raises the question as to whether the intervention is truly ‘collaborative care’ in the absence of joint interaction between professionals, but beyond conceptual fidelity, it also raises an empirical question about whether joint interaction is a necessary component of collaborative care for improving outcomes. Overcoming existing cultural and organizational barriers to inter-professional working around mental and physical health is likely to be extremely difficult, and professionals may need to be convinced that this joint working is in fact necessary for the model to be effective. Future studies of collaborative care should first assess the impact of inter-professional working on health outcomes, and then explore patient experience to determine if greater professional interaction is perceived by patients as improving the quality of care. In the presence of complex mental-physical multi-morbidity, there is a growing need to understand patient preferences and priorities for treatment and self-management [23]. New approaches to multi-morbidity such as those advocating ‘minimally disruptive medicine’ emphasize the burden of treatment carried by patients with multiple conditions [24]. It should not be assumed that increasing interactions between different professionals and patients reduces the burden of care, as patients may view such interactions as further adding to the complexity of illness management, particularly when typical patterns of service provision (such as the division of mental and physical treatments) are disrupted. Exploring patient experiences and priorities for managing multiple conditions is therefore a crucial focus for future research.

This naturalistic pilot study exposed the kind of adaptations made in practice to prototypical models of collaborative care, leading to questions about the necessary mechanisms of collaboration and their impact on outcomes that can now be tested under trial conditions. This demonstrates the value of naturalistic pilot phases in the development of complex interventions, as barriers to adoption of the intervention in practice can be identified and subsequently monitored within randomized controlled trials to rigorously evaluate their impact on effectiveness. For example, the COINCIDE trial will collect activity logs from professionals to assess the degree of joint working and the relationship of this to patient satisfaction and outcomes.

In this study, NPT was employed to examine the extent to which collaborative care can be ‘normalized’ under routine conditions. The study findings show that integrating innovative models of care into routine practice can lead to elements of that innovation that clash with existing norms being neglected, and exposes the tensions inherent in such research which necessarily aims to disrupt, or ‘denormalize,’ certain ways of working in order to introduce new ones. Similar studies of innovative models for LTC management in primary care have shown the difficulties that exist when trying to modify established roles in order to improve care [25]. Other models of implementation, such as the PARIHS framework [26] emphasize the need for facilitation through leadership to bring about such changes. Future qualitative studies should explore the role of clinical leaders, in this case GPs and IAPT supervisors, in supporting collaboration across mental and physical health boundaries. Given that collaborative care for mental-physical multi-morbidity involves complex interactions across primary care both vertically (from front line primary care workers to GPs and service leaders) and horizontally (across mental and physical health services), models of healthcare that consider both designated and distributed leadership may be particularly useful [27].

Bridging the translational gap between research and practice may depend on bridging the gap in our understanding of implementation from models that describe organizational change and adoption of novel ways of working across to those which emphasize the need to embed change within existing structures. Programs such as CLAHRC are uniquely placed to contribute to such understanding and future work should further explore the tensions between the need for innovation and integration in primary care.

## Conclusions

In conclusion, our analysis demonstrated that implementation of collaborative care in routine settings can be hindered by:

1. Lack of engagement with the organizational aspects of the model, which may be neglected even if the patient level factors of continuity of care and holistic care are valued.

2. Pre-existing structures and norms that emphasize division of labor between the provision of physical and mental healthcare.

Future research should address the level of collaboration necessary to deliver improved outcomes, and explore the patient experience of collaborative working across mental and physical health services.

Primary care is the focal point for management of LTCs in the UK and also manages the majority of patients with health problems. Primary care therefore offers a key opportunity to address the overlap between these conditions and provide more integrated care for patients with mental-physical multi-morbidity [4]. Taking advantage of this opportunity requires bridging the translational gap to implement improved ways of working into the complex reality of day to day primary care, but questions remain regarding how to organize such services in ways that are acceptable to health professionals, and indeed whether such models of multi-morbidity care are acceptable to patients themselves.

## Abbreviations

IAPT: Improving access to psychological therapies; NPT: Normalization process theory; LTCs: Long-term conditions; CLAHRC: Collaborative leadership in applied health research and care; PWP: Psychological wellbeing practitioner; PN: Practice nurse.

## Competing interests

The authors declare that they have no competing interests.

## Authors’ contributions

PC led the pilot phase. PC and CCG contributed to the design of the training program. SK prepared the manuscript. SK, CCG, NC and PC designed the study and revised the manuscript. NC, IA, CK and HT collected the data. All authors contributed to analysis and have given final approval for the manuscript to be published. All authors read and approved the final manuscript.

## Supplementary Material

Additional file 1 Normalization Process Theory analysis. Click here for file

## References

[B1] Kendrick T Dowrick C McBride A Howe A Clarke P Maisey S Management of depression in UK general practice in relation to scores on depression severity questionnaires: analysis of medical record data BMJ 2009 338 mar19 1 b750 b750 10.1136/bmj.b750 19299475

[B2] Bayliss EA Steiner JF Fernald DH Crane LA Main DS Descriptions of Barriers to Self-Care by Persons with Comorbid Chronic Diseases Ann Fam Med 2003 1 1 15 21 10.1370/afm.4 15043175 PMC1466563

[B3] Boyd CM Fortin M CMFC MDM Future of Multimorbidity Research: How Should Understanding of Multimorbidity Inform Health System Design? Public Health Rev 2010 32 2 451 74

[B4] Mercer SW Gunn J Bower P Wyke S Guthrie B Managing patients with mental and physical multimorbidity BMJ 2012 345 sep03 1 e5559 e5559 10.1136/bmj.e5559 22945951

[B5] Van Korff M Katon W Unutzer J Wells K Wagner E Improving Depression Care Barriers, Solutions, and Research Needs J Fam Pract 2001 50 6 Available from: http://www.jfponline.com/pages.asp?aid=2253 11401751

[B6] Bodenheimer TWE Improving primary care for patients with chronic illness JAMA 2002 288 14 1775 9 10.1001/jama.288.14.1775 12365965

[B7] Gunn J Diggens J Hegarty K Blashki G A systematic review of complex system interventions designed to increase recovery from depression in primary care BMC Health Serv Res 2006 6 1 88 10.1186/1472-6963-6-88 16842629 PMC1559684

[B8] Archer J Bower P Gilbody S Lovell K Richards D Gask L Collaborative care for depression and anxiety problems. Cochrane Database of Systematic Reviews [Internet] 2012 John Wiley and Sons, Ltd [cited 2012 Nov 20]. Available from: http://onlinelibrary.wiley.com/doi/10.1002/14651858.CD006525.pub2/pdf/standard 10.1002/14651858.CD006525.pub2 PMC11627142 23076925

[B9] Gilbody S Whitty P Grimshaw J Thomas R Educational and organizational interventions to improve the management of depression in primary care JAMA 2003 289 23 3145 10.1001/jama.289.23.3145 12813120

[B10] Katon WJ Von Korff M Lin EHB Simon G Ludman E Russo J The Pathways Study: a randomized trial of collaborative care in patients with diabetes and depression Arch Gen Psychiatry 2004 61 10 1042 9 10.1001/archpsyc.61.10.1042 15466678

[B11] Katon WJ Seelig M Population-based care of depression: team care approaches to improving outcomes J Occup Environ Med 2008 50 4 459 67 10.1097/JOM.0b013e318168efb7 18404019

[B12] Katon W Unutzer J Collaborative Care Models for Depression: Time to Move From Evidence to Practice Arch Intern Med 2006 166 21 2304 6 10.1001/archinte.166.21.2304 17130381

[B13] Gilbody S Bower P Fletcher J Richards D Sutton AJ Collaborative care for depression: a cumulative meta-analysis and review of longer-term outcomes Arch Intern Med 2006 166 21 2314 21 10.1001/archinte.166.21.2314 17130383

[B14] Coventry PA Lovell K Dickens C Bower P Chew-Graham C Cherrington A **Collaborative Interventions for Circulation and Depression (COINCIDE): study protocol for a cluster randomized controlled trial of collaborative care for depression in people with diabetes and/or coronary heart disease.** Trials 2012 13 1 139 10.1186/1745-6215-13-139 22906179 PMC3519809

[B15] Department of Health Talking therapies: a 4 year plan of action [Internet] 2011 Available from: https://www.gov.uk/government/uploads/system/uploads/attachment_data/file/213765/dh_123985.pdf

[B16] Medical Research Council A Framework for development and evaluation of RCTs for Complex Interventions to Improve Health [Internet] 2011 Available from: http://www.mrc.ac.uk/Utilities/Documentrecord/index.htm?d=MRC003372

[B17] May C Finch T Mair F Ballini L Dowrick C Eccles M Understanding the implementation of complex interventions in health care: the normalization process model BMC Health Serv Res 2007 7 1 148 10.1186/1472-6963-7-148 17880693 PMC2089069

[B18] Gunn JM Palmer VJ Dowrick CF Herrman HE Griffiths FE Kokanovic R Embedding effective depression care: using theory for primary care organisational and systems change Implement Sci 2010 5 1 62 10.1186/1748-5908-5-62 20687962 PMC2925331

[B19] Gask L Bower P Lovell K Escott D Archer J Gilbody S What work has to be done to implement collaborative care for depression? Process evaluation of a trial utilizing the Normalization Process Model. Implement Sci 2010 5 15 10.1186/1748-5908-5-15 20181163 PMC2829490

[B20] Franx G Oud M de Lange J Wensing M Grol R Implementing a stepped-care approach in primary care: results of a qualitative study Implement Sci 2012 7 1 8 10.1186/1748-5908-7-8 22293362 PMC3292960

[B21] Chaney E Rubenstein L Liu C-F Yano E Bolkan C Lee M Implementing collaborative care for depression treatment in primary care: A cluster randomized evaluation of a quality improvement practice redesign Implement Sci 2011 6 1 121 10.1186/1748-5908-6-121 22032247 PMC3219630

[B22] Wells KB Sherbourne C Schoenbaum M Duan N Meredith L Unützer J Impact of Disseminating Quality Improvement Programs for Depression in Managed Primary Care JAMA 2000 283 2 212 220 10.1001/jama.283.2.212 10634337

[B23] Mangin D Heath I Jamoulle M Beyond diagnosis: rising to the multimorbidity challenge BMJ 2012 344 jun13 2 e3526 e3526 10.1136/bmj.e3526 22695898

[B24] May C Montori VM Mair FS We need minimally disruptive medicine BMJ 2009 339 aug11 2 b2803 b2803 10.1136/bmj.b2803 19671932

[B25] Peters S Wearden A Morriss R Dowrick C Lovell K Brooks J Challenges of nurse delivery of psychological interventions for long-term conditions in primary care: a qualitative exploration of the case of chronic fatigue syndrome/myalgic encephalitis Implement Sci 2011 6 1 132 10.1186/1748-5908-6-132 22192566 PMC3259041

[B26] Kitson AL Rycroft-Malone J Harvey G McCormack B Seers K Titchen A Evaluating the successful implementation of evidence into practice using the PARiHS framework: theoretical and practical challenges Implement Sci 2008 3 1 1 10.1186/1748-5908-3-1 18179688 PMC2235887

[B27] Best A Greenhalgh T Lewis S Saul JE Carroll S Bitz J Large-system transformation in health care: a realist review Milbank Q 2012 90 3 421 56 10.1111/j.1468-0009.2012.00670.x 22985277 PMC3479379

