# Supplementary material for: Better together? a naturalistic qualitative study of inter-professional working in collaborative care for co-morbid depression and physical health problems
Source: Implement Sci. 2013 Sep 20;8:110. doi: 10.1186/1748-5908-8-110 (PMC3848572; doi:10.1186/1748-5908-8-110)
Supplement: Additional file 1 — Normalization Process Theory analysis. [file 1748-5908-8-110-S1.doc]

Additional file1 Normalization Process Theory analysis.

**A1: Coherence**

***Intervention provides holistic care by providing both mental and physical health care – although separately:***

‘You’re doing joined up work, treating somebody holistically. I’m doing the medical side of it and they’re doing the mental health side.’ PN09

‘I think it means that it’s different areas of the health service working together …mental health and physical health they’re treated together so that the person is treated as a whole person, not just separate parts’ PWP05

**A2: Cognitive Participation**

***Assumption that intervention will better divide physical/mental health work:***

‘It frees us up, the Doctors and the Nurses, to concentrate perhaps more on the physical reviews, physical conditions’ PN01.

***Preference for physical/ mental health work to remain with the other professional:*** ‘I find it quite hard work, personally, to delve into some of these underlying problems, I haven’t got the knack as yet of how to shut them up.’ PN09

‘You are trying to work with somebody who is depressed and you are talking about lethargy but the reason for that might be because of chronic pain… I wouldn't necessarily know how to or have that much time to do that.’ PWP01

**B2: Cognitive Participation**

***Reluctance to arrange joint sessions:***

‘I don’t want them to think I’m nosing in I want them to feel their time with her is protected’ PN05

‘The First Step practitioner, will have a different relationship, with the patient, than I will, although we’re still working towards the same aim, so, meeting together, … would be a bit intimidating, for the patient’ PN10

***Lack of formal/top down support for case management role:***

‘A collaborative meeting … is absolutely key… but that wasn't built in at all, I suggested that …because I'm not prepared to take referrals in a corridor in a GP surgery’ PWP01

‘In practice, I don’t feel like we are encouraged to be the case manager that they talk about in theory …, I think management would say, well, the client is responsible for themselves.’ PWP04

**B1:Coherence**

***Uncertainty over case management role***:

‘I don’t think I know what a Case Manager is.’ PWP05.

‘I don’t remember signing up to have to educate GPs, … but I’m also aware that it’s part of my role to spread the word about what the service does. But you can only try so hard.’ PWP02

***Practices do not understand collaborative model:*** ‘Trying to explain what we’re doing… filtering that message across towards the practice nurses, and then further up to GPs…it’s like Chinese whispers sometimes and people get confused.’ PWP06

**C1. Collective Action**

***Collaborative system not used or valued****:* ‘Everyone seems to agree in principal but the actual day to day stuff actually seems to be going further away. The GPs don’t refer to us, they’re handing out leaflets for self referral, so that’s further away from collaboration … they think that we’re competent to do separately. It’s not seen as a valued… that referral doesn’t seem like a necessity… other things have the same outcome in terms of that patient gets referred to the service but without [GPs] having to lift a pen’PWP03

***PWPs not integrated with the practice****:* ‘I asked to go to the practice meeting to do a five minute thing, I said I’d be in and out and I was declined that… I just keep turning up like a bad penny every time I’m meant to be there, hanging around at the sort of communal bits, bumping into people’ PWP02

‘There’s very much an ‘us and them’ feeling, within the surgery… the surgery staff and admin staff are so used to other practitioners being in…we’re not really noticed’ PWP04

**C2. Reflexive Monitoring**

***PWPs and PNs differ in perception of need for monitoring:*** ‘She’s got a tray, an input tray, upstairs like all of us, so, you know… if she wants to contact us, she’s got my mobile number.’ PN012

‘I phoned a nurse one day and it’s taken days and days to get a response, because they’re just too busy. .. So if you don’t get them face to face, you don’t get them at all.’ PWP02

***PWPs isolated - unclear how to feedback to practice:*** ‘I wouldn’t know who to feedback to. I don’t know who that would be. I don’t see the GPs. It’s fractured.’ PWP03

‘I don’t think there’s any point, really, if they’re getting helped, I don’t need to know the nitty gritty of it’ PN10

Patient-level work

Service-level work
